# Supplementary material for: The World Health Organization (WHO) Integrated Care for Older People (ICOPE) Framework: A Narrative Review on Its Adoption Worldwide and Lessons Learnt
Source: Int J Environ Res Public Health. 2022 Dec 22;20(1):154. doi: 10.3390/ijerph20010154 (PMC9819593; doi:10.3390/ijerph20010154)
Supplement: Supplementary file 1 [file ijerph-20-00154-s001.zip › ijerph-2023514-supplementary.pdf]

Supplementary File S1. Search Strategy for each database

PubMed

#1	"World Health Organisation"[Title/Abstract] OR "World Health Organization"[Title/Abstract] OR "WHO"[Title/Abstract]	2,579,296
#2	"Integrated Care for Older People"[Title/Abstract] OR ICOPE[Title/Abstract]	87
#3	#1 AND #2	35
#4	#3 AND ("2017/10/31"[Create Date] : "2022/03/31"[Create Date])	32

Embase (Ovid)

Database: Embase <1974 to 2022 April 12>
Search Strategy:

----

1 ("World Health Organisation" or "World Health Organization" or "WHO").ab,ti. (3768331)
2 ("Integrated Care for Older People" or ICOPE).ab,ti. (89)
3 1 and 2 (34)
4 limit 3 to dc=20171031-20220331 (29)

Cumulative Index of Nursing and Allied Health Literature (EBSCO)

S1	TI ("World Health Organisation" or "World Health Organization" or "WHO") OR AB ("World Health Organisation" or "World Health Organization" or "WHO")	871,988
S2	TI ("Integrated Care for Older People" or ICOPE) OR AB ("Integrated Care for Older People" or ICOPE)	70
S3	S1 AND S2	24

S4	S1 AND S2 (Limiters - Published Date: 20171001-20220331)	19

Global Health (EBSCO)

S1	TI ("World Health Organisation" or "World Health Organization" or "WHO") OR AB ("World Health Organisation" or "World Health Organization" or "WHO")	481,317
S2	TI ("Integrated Care for Older People" or ICOPE) OR AB ("Integrated Care for Older People" or ICOPE)	9
S3	S1 AND S2	4
S4	S1 AND S2 (Limiters - Publication Year: 20171001-20220331)	4

Conference proceedings of the International Conference of Frailty and Sarcopenia Research (ICFSR)

#1	"World Health Organisation"[Title/Abstract] OR "World Health Organization"[Title/Abstract] OR "WHO"[Title/Abstract]	2,579,296
#2	"Integrated Care for Older People"[Title/Abstract] OR ICOPE[Title/Abstract]	87
#3	#1 AND #2	34
#4	"International Conference of Frailty and Sarcopenia Research" OR ICFSR	30
#5	#3 AND #4	1

Google Scholar

("World Health Organisation" or "World Health Organization" or "WHO") AND ("Integrated Care for Older People" or ICOPE)

Custom Range: 2020-2022

Scopus

TITLE-ABS-KEY (("World Health Organisation" OR "World Health Organization" OR "WHO") AND ("Integrated Care for Older People" OR icope)) AND (LIMIT-TO (PUBYEAR , 2022) OR LIMIT-TO (PUBYEAR , 2021))

ProQuest Dissertations and Theses Global

("World Health Organisation" OR "World Health Organization" OR "WHO") AND ("Integrated Care for Older People" OR ICOPE)

Select option: 'Anywhere'

Entered date: 2017-10-31 - 2022-03-31

Global Index Medicus

("World Health Organisation" or "World Health Organization" or "WHO") AND ("Integrated Care for Older People" or ICOPE)

Reports of the Clinical Consortium for Healthy Ageing for Implementation Initiatives Across Member States

Hand searching of WHO reports

The World Health Organisation Institutional Repository for Information Sharing (IRIS)

("Integrated Care for Older People" OR ICOPE)

Supplementary File S2

Table S1. Additional information from data extraction (n=29)

AMICOPE: Aptitude Multi-domain group-based intervention to improve and/or maintain Intrinsic Capacity in Older People; **IC**: Intrinsic capacity; **MAPT:** The Multidomain Alzheimer Preventive Trial

Reference	Study objective	Study population, sample size, inclusion/exclusion criteria	Additional stakeholders	Additional comments on adoption and application of ICOPE
Alekhina et al 2021	To improve the quality of life of older citizens, as well as to use digital technology to early detect diseases, prolong ability to be active and increase life expectancy.	Patients aged 65 years and above.	Not specified.	NA
Blancafort Alias et al (2021)	To develop of a multi-domain group-based intervention for older people living in the community, which aims to improve and/or maintain intrinsic capacity by means of promoting physical activity, healthy nutrition, and psychological wellbeing.	Older people with losses in mobility, nutritional and/or psychological domains of intrinsic capacity, and without cognitive decline, visual impairment or hearing loss, living in the community, and recruited or referred from primary care and community settings.<br><br>Participants had to be able to attend the intervention on their own. Authors focused on a phase previous to frailty with a lower prevalence of diseases and disability, and without losses in the sensorial domain.	The working group that reviewed the general scope of the intervention and basic components of the multi-domain intervention included professionals from different fields (geriatrics, neuropsychology, nutrition, pharmacy, primary care, occupational therapy, physiotherapy, public health, social work, sociology of health, sports medicine and sports science research).<br><br>Stakeholders who provided feedback to define specific contents and procedures included health and social care professionals, public administration, civic organizations (older people's associations, patients' associations, women's associations, non-profit private foundations) and entrepreneurs of the silver economy	This study represents the first stage of the UK Medical Research Council framework for developing and evaluating a complex intervention. The next step should be carrying out a feasibility study for the AMICOPE intervention and, at a later stage, assessing the effectiveness in a randomized controlled trial.
Cheng et al (2021)	The objective was to use the ICOPE as a screening tool to identify functional impairment, and to explore the association of geriatric functional impairment and	Community-dwelling elderly aged at least 65 years with hypertension, diabetes or dyslipidaemia, and elderly aged at least 75 years		NA

	hypertension, diabetes, dyslipidaemia in the community-dwelling elderly.	n=457<br><br>Inclusion criteria:<br>Age at least 65 years with hypertension, diabetes, dyslipidemia, or age at least 75 years, and signed informed consent.<br><br>Exclusion criteria:<br>Younger than 65 years, or younger than 75 years with no hypertension, diabetes, or dyslipidaemia.		
de Souto Barreto et al (2021)	To implement a function-centred healthcare pathway for aging. The INSPIRE program aimed to implement the ICOPE recommendations from the WHO. ICOPE recommends to focus the care of older individuals according to the five domains of intrinsic capacity (function-centred approach), and INSPIRE would allow the authors to go beyond the simple implementation of ICOPE, by fostering investigations on both the biological changes related to healthy aging and the development of new technologies and metrics enabling the overtime monitoring of functions during aging. It will also identifying biomarkers of aging. INSPIRE will provide clinical and digital data as well as biospecimens from both human and animals, to foster the identification and validation of (a set of) biomarkers of aging.	Older individuals.	Local (Regional Health Agency in the Region Occitanie) and national (French Ministry of Health) health authorities.	By implementing the ICOPE model in routine care, the INSPIRE program will be contributing to the change in the care approach to aging, replacing the current disease-centred model by a function-centred healthcare pathway. Furthermore, by following them overtime through the ICOPE app, this real-life population may constitute a pool of potential participants for the activities of the INSPIRE program (e.g., feasibility of the remote monitoring of IC levels, validation of biomarkers, etc).
González-Bautista et al (2021)	To apply an adaptation of the screening tool ICOPE Step 1 to retrospectively estimate the frequency of priority conditions associated with declines in intrinsic capacity.<br><br>MAPT was a 3-year randomized controlled trial on the effect of a multidomain intervention (nutritional and	Community-dwelling older adults aged 70 years or older without dementia at baseline.<br><br>n= 759<br><br>Inclusion criteria for the MAPT study:<br>At least one of three conditions: a) spontaneous memory complaint	Not specified.	NA

	physical activity counselling, cognitive training, and annual preventive consultations for the management of cardiovascular risk factors and the detection of functional impairments) with and without supplementation of omega-3 polyunsaturated fatty acids (PUFA) on the prevention of cognitive decline among community-dwelling adults aged 70 years and older.	expressed to their physician, b) limitation in one instrumental activity of daily living (IADL), or c) slow gait speed (≤0.8 m/s).<br><br>Exclusion criteria: Comprised participants with a Mini-Mental State Examination (MMSE) score < 24, diagnosis of dementia, the limitation for any of the basic activities of daily living, and those taking PUFA supplements at baseline.		
Gonzalez-Bautista et al (2021)	To use the ICOPE Step 1 tool to test if the older adults who failed recalling the name of the weekday or had a higher number of mistakes in the word recall were at higher risk of mild cognitive impairment (MIC) or dementia.	As above	As above	NA
Gonzalez-Bautista et al (2021)	To evaluate the ICOPE screening tool's ability to identify people at risk of developing frailty and disability in basic (ADL) and instrumental (IADL) activities of daily living among community-dwelling older adults.	As above	As above	NA
Gutierrez-Robledo et al 2021	To describe the levels of IC and those factors related to its decline in Mexican older adults, using data from the Mexican Health and Aging Study (MHAS).	Older adults aged 50 years and older in the Mexican population.<br><br>n= 14 779<br><br>The MHAS wave 2015 had 14,779 persons, of which 12 459 (84.3%) had complete information for all IC domains.	Not specified.	Variables from the corresponding sets of MHAS questionnaires were selected to construct a screening tool. For each of the domains, a score of 1 was assigned to a positive screening result. Scores were added to reach a single score, ranging from 0 to 6. Further grouping was done according to this score into four groups considering the distribution of the variable: no affected domain (0), one affected domain, two or three, and four to six affected domains. The objective of categorizing in this way was to show the number of affected domains rather than summarize in a continuous variable. Regarding cognition, verbal recall memory and orientation tests were used; having any of these wrong was considered a positive screening result. For the psychological domain, two questions from the MHAS symptoms of depression assessment were used. Self-rated vision and hearing were used to assess these domains; screening was considered positive when the answer was fair, poor, or legally blind/deaf. Regarding vitality, weight loss and anorexia were

				evaluated as proxies for this domain. Screening for mobility problems was positive if the individual responded “yes” to a question about having difficulty walking several blocks or climbing several flights of stairs without resting..
Guyonnet et al (2020)	1) To explore and identify a set of biomarkers of aging, age-related diseases and IC evolution;<br>2) To test the feasibility and acceptability of a new app for smartphone and tablet developed to monitor the six IC domains (locomotion, cognition, vision, hearing, vitality/nutrition, psychological status), according to the WHO recommendations<br>3) To explore the development of digital markers of aging	Community dwelling adults (20 years old and above)<br><br>Frail adults from residential homes, long-term care facilities and post-acute and<br>rehabilitation facilities<br><br>Older adults aged 20 years and above.<br><br>Exclusion criteria: people having a severe disease compromising life expectancy at 5 years (or at 1 year for subjects living in nursing homes) and people deprived of their liberty by administrative or judicial decision, or under guardianship	Clinical Research Centre (CRC) of the Gerontopole.	
Lafont et al 2021	Not specified.	Independent older adults aged 60 years and over.	Not specified.	Digital tools have been developed to facilitate the assessment and follow-up of persons in this care project.
Liu et al (2021)	To analyse the predictive value of intrinsic capacity measured by ICOPE screening tool on the incidence of functional decline and falls in a superior quality senior community and compare its value with frailty.	Active, independent older adults living in the compound senior community.<br><br>n=227.<br><br>The inclusion criteria were: (1) aged over 75 years and (2) lived in the CCRC active area from June to August 2018.<br><br>The exclusion criteria were: (1) severe cognition impairment diagnosed by neurologist and (2) acute heart failure, myocardial infarction within 3 months, acute exacerbation of chronic obstructive pulmonary disease, and acute lung infection	Peking Union Medical College Hospital (PUMCH), Taikang Yanyuan continuing care retirement community (CCRC).	To construct a standard index of intrinsic capacity, taking the weight of influence of each domain into account, and examine the effect of person-centred intervention based on intrinsic capacity.
Ma et al (2020)	To determine if the WHO ICOPE screening tool can identify adults with	Healthy participants without acute illness and aged ≥50 years.	Not specified.	NA

	poor physical and mental function in a Chinese population.	n=376<br><br>The exclusion criteria were participants who suffered from acute heart failure, acute infection, acute cerebrovascular disease, severe cardiac, liver or kidney dysfunction, dementia, Parkinson's disease, and mental illnesses or were unable to provide informed consent because of aphasia, deafness, and blindness.		
Ma et al (2021)	To examine the plasma NT-proBNP levels in participants with decreased intrinsic capacity.	Healthy participants without acute illness and aged ≥60 years.<br><br>n=283<br><br>The exclusion criteria were suffered from heart failure, acute diseases, severe cardiac, liver or kidney dysfunction, Parkinson's disease, mental illnesses, and dementia		NA
Mathieu et al 2021	Not specified.	Older adults with age not specified.	Not specified.	NA
Mathur et al 2021 (preprint)	To demonstrate the feasibility of the community-based screening of older people using WHO ICOPE screening tool in a rural Indian population.	n= 451<br><br>Geriatric people aged 60 years and above were included in the study through a home-based survey.<br><br>Mean age of 68.36 (ranged from 60 to 98 years), 54.5% males	Not specified.	As per the WHO ICOPE screening tool, cognitive decline was determined if there was difficulty or failure in answering to questions related to orientation of time and place or recalling words; decline in mobility was assessed through the chair rise test; and malnutrition was assessed through recent weight loss or loss of appetite. Sensory loss was determined through the assessment of visual and hearing impairment and depressive symptoms were assessed through scoring patients' perception of feeling down, depressed, hopeless and lack of interest in doing things over past 2 weeks.<br><br>The components of the intrinsic capacity were scored either 0 – representing decline or 1 – representing no decline.
Pages et al 2022	To explore the association between deficits in IC domains as measured by an adapted Step 1 screening tool and healthcare costs at both short-term (primary objective) and	Participants were aged 70 years or older, and community-dwelling without dementia at baseline.	Not specified.	A deficit (binary: yes/no) in IC was determined for each domain. Persistent impairment of IC was defined per domain as having IC deficits in at least two time-points (consecutive or non-consecutive) over the follow-up, participants for whom it was

	long-term (secondary objectives) among older patients.	n= 693<br><br>Inclusion criteria: Spontaneously complained about their memory, or limitation in one instrumental activity of daily living, or usual gait speed ≤ 0.8 m per second or more than 5s in the 4 m walk test.<br><br>Exclusion criteria: a Mini-Mental State Examination (MMSE) score lower than 24, diagnosed dementia,<br>and current supplementation with omega 3 at baseline		not possible to ascertain the status of persistent impairment (eg, due to missing data on IC domains) were excluded from analysis on persistent impairment.
Piau et al 2020	To illustrate the potential contribution "digital biomarker" to the need for intrinsic capacity monitoring.<br><br>To explore and identify biomarkers of aging and IC evolution through annual biological, clinical, and digital data over 10 years, and therefore through both so-called conventional biomarkers and digital biomarkers.<br><br>To better understand the evolution of IC over time, followed by a web-application for IC self-monitoring, the ICOPE MONITOR Smartphone-app.	20 years old and above.	Not specified	To implement INSPIRE ICOPE-CARE program and launched CART-France digital biomarkers cohort where a subgroup of 100 INSPIRE-T participants will be monitored by ambient and wearable sensors at home (e.g., infrared sensors on the ceiling, bed mat) over a long period of time. This will allow remote and continuous monitoring of IC trajectories and detect subtle changes that are not readily detected with conventional methods.<br><br>If the time gap between two self-assessments is relatively long (4 months) and if the measurement is not yet sensor-mediated, this type of organization paves the way for the wider use of digital biomarkers for IC monitoring (e.g., automatic and unobtrusive measurement of transfers from sitting to standing by an accelerometer throughout the day).
Prince et al 2021	The objectives were to (1) determine the prevalence of intrinsic capacity overall, and in its several domains, clarifying the proportion of individuals likely to be identified with significant declines in IC (DIC), and hence to be the target population for the ICOPE intervention; (2) characterise the target group, and compare them with others, for burden of disease (behavioural and lifestyle risk factors, morbidities, and disability) and use of	Older people aged 65 years and over living in geographically defined catchment areas in 8 countries. Baseline population-based surveys were carried out between 2003 and 2007, and follow-up assessments between 2008 and 2010.<br><br>n= 17,031<br><br>Participants were surveyed at baseline in the 12 sites in 8 countries. Mean age	The 10/66 research group had links with the national Alzheimer's Associations and formed intersectoral links with other relevant NGOs e.g., HelpAge International, Save the Children (cross-generational effects), Oxfam (economic impact of aged care, and poverty reduction).	Seven ICs were evaluated at baseline, linked to the concepts and, where possible, the operationalisations currently within the WHO ICOPE draft<br>guidance on comprehensive assessment. For each IC, a threshold was applied to determine whether the individual has retained capacity or experienced DIC. The decline was inferred from a single assessment, and not directly observed. As such, long-term stable impairment could have been misclassified as age-related decline. The ICs include:

	healthcare services; (3) establish the extent of the overlap of DIC with frailty and dependence (needs for care), and the concentration of burden indicators in each of these 3 groups when defined hierarchically; and (4) clarify the prospective association of DIC with incident dependence and mortality.<br><br>It was a secondary analysis of data collected from the 10/66 Dementia Research Group (10/66 DRG) population-based baseline and incidence wave surveys in 12 catchment areas in 8 LMICs in Latin America, China, and India.	varied from 71.3 and 76.3 years and was higher in urban than rural areas and in more-than less-developed sites. 62.4% were females.<br><br>Eligible participants were all those normally resident (in that site) and aged 65 years or over on the defined census date.		(1) Neuromusculoskeletal capacity: Walking speed was assessed using a timed walking test (5 metres at usual speed, turn, and return to the starting point); those completing the test in less than 16 seconds were considered to have capacity (allowing 3 seconds to make the turn, this corresponds to a walking speed of >0.8 m/s).<br><br>(2) Vitality: Nutrition was considered adequate if the older person did not report weight loss of 4.5 kg in the last 3 months, and if their mid-upper arm circumference was measured to be 22 cm; this cut-point is used in the Mini Nutritional Assessment (MNA) to identify the most severe level of undernutrition.<br><br>(3) Sensory capacity (visual impairment): Vision was considered adequate when the older person did not report 'eyesight problems' that interfered with their activities to at least some extent, and when they were not identified by the interviewer as being functionally blind.<br>(4) Sensory capacity (hearing loss): Hearing was considered adequate when the older person did not report 'hearing problems or deafness' that interfered with their activities to at least some extent, and when they were not identified by the interviewer as being profoundly deaf.<br><br>(5) Cognitive capacity: Cognitive function was assessed using the Community Screening Instrument for Dementia (CSI-D) COGSCORE, which tests multiple domains of cognitive function and has been found to have robust cross-cultural measurement properties in the 10/ 66 DRG study sites; those who scored 29.5 were considered to have cognitive capacity, with scores below that threshold identifying 'probable dementia'.<br><br>(6) Psychological capacity: Psychological capacity was considered to be present if participants endorsed 3 or fewer of the 12 depression symptoms covered in the EURO-D depression scale; in previous analyses this cut-point identified individuals with significant impairment of health-related quality of life, although a higher cut-point is better for identifying clinical cases.

				(7) Continence: Incontinence (urinary, faecal, or both) was established from informant report only, and capacity was maintained if none of these were reported. Continence is not currently a primary focus of the ICOPE comprehensive assessment tool, although guidelines for the assessment and management of incontinence have been prepared by the guideline development group.
Sanchez-Rodriguez et al (2021)	The objectives were:<br>1) to assess the relationship between intrinsic capacity assessed with the ICOPE Apps and the incidence of frailty in community-dwelling older adults during one year follow up; 2) to assess the association between intrinsic capacity measured by the ICOPE App and the ICOPE Monitor at baseline and the risk of functional decline, mortality, incidence of pre-frailty, falls, institutionalization, and loss of quality of life.	Older individuals. Community-dwelling adults, ≥65-year-old living at home.<br><br>n=600.<br><br>Exclusion criteria: Inability to use the ICOPE Apps or communicate by telephone/video-call for any reason (cognitive or limited access to technologies like telephone/video-call) OR being considered frail at baseline (defined as having a Rockwood's clinical frailty scale, CFS score ≥4).	Not specified	The results in the two Apps provide different approaches to IC. First, a global binary decision (IC decline or not) is directly provided by the ICOPE App (IC decline or not). Second, a binary decision of each one of the 5 domains per separate is provided directly by the ICOPE Monitor (each domain decreased or not); third, the binary decision obtained in the 5-domains could be combined as a score which might be just a count of IC declines, e.g. if we have 3 domains with decline among the 5 domains, the score would be 3/5. For purpose of this analysis, the second approach, a binary decision of each one of the 5 domains per separate provided directly by the ICOPE will be selected; the other two options would be studied as part of further secondary analysis.
Smith et al (2018)	The objective was to identify and intervene on those older adults who are at under or over nutrition<br>risk via the primary care office pathway.	Older persons with risk of becoming or is poorly nourished.	Not specified.	NA
Takeda et al (2020)	The main objective is to create an innovative research platform combining biological, clinical (including imaging data) and digital data from patient in primary care.<br><br>The secondary objectives (summarised):<br>a) to create a web platform to track subjects remotely with collection of lifestyle and clinical data (especially on intrinsic capacity);<br>b) to examine the implementation outcomes for screening, evaluation and monitoring of IC;<br>c) to study the trajectory of different domains of intrinsic capacity over time	30 years old and above.	Toulouse regional team for ageing and prevention	A final digital cohort will be followed by the digital ICOPE application tailored to the study's needs. Aims to deploy the ICOPE approach at the national level.

	(natural history), their interactions, predictors and the causes of their decline<br>d) to discover markers that could be outcomes for clinical trials in the field of Geroscience;<br>e) to explore the development of digital markers of ageing through the ICOPE app			
Tavassoli et al (2021)	The main objective of the INSPIRE ICOPE-CARE program is to implement, in clinical practice at a large scale, the WHO ICOPE program in the Occitanie region, in France, to promote healthy aging and maintain the autonomy of seniors using digital medicine. Secondary objectives are to explore the acceptability of the INSPIRE ICOPE-CARE program by both older adults and health care professionals, as well as to examine the use of new digital tools in the evaluation and monitoring of intrinsic capacity.	Independent seniors aged 60 years and over. 950 seniors have been assessed.	Ecosystem of organisations, government ministries, and town halls.	The INSPIRE ICOPE-CARE program plans to screen and monitor the intrinsic capacity of 200,000 older people in Occitanie region within five years and promote preventive actions, instead of only punctual, curative ones. The French Presidential Plan Grand Age aims to largely implement the WHO ICOPE program in France following the experience of the INSPIRE ICOPE-CARE program in Occitania.
Valliant-Ciszewicz et al 2021	Not specified	Dementia patients and their caregivers<br><br>n=14	Geronto-psychologist, Physician	NA
Won et al (2021)	To identify how the ICOOP_Frail could contribute to the WHO ICOPE by reviewing the associations between the two programs.	Not described.	National Evidence-based Healthcare Collaborating Agency in Korea, Gerontologic epidemiology at Kyung Hee University.	The possible next steps were to use this study as a reference for future ICOPE implementation studies in Korea. In the future, volunteers or public health workers in the community would be invited to serve as health coaches.
Yu et al 2022	The primary objective was to determine prevalence and distribution of IC impairments. The secondary objective was to examine the longitudinal associations of the number of impaired IC domains with the incidence of polypharmacy, incontinence, poor/fair self-rated health and instrumental activities of daily living (IADL) difficulty among community-dwelling older Chinese people.	n=10,007<br><br>Inclusion criteria: aged 60 years and older and community-dwelling.<br><br>Exclusion criteria: Not specified	80 community elderly centres	Six IC domains were assessed:<br>1) Cognition - 5-item Abbreviated Memory Inventory for Chinese (AMIC). Those scored ≥3 were considered as having an impairment in cognitive domain<br>2) Locomotion - Item 2 and 3 of 5-item FRAIL scale. Item 2: Resistance (Do you have any difficulty walking up 10 steps alone without resting and without aids?). Item 3: Ambulation (Do you have any difficulty walking several hundred yards alone and without aids?). Those who answered "Yes" to any of the two items were considered as having an impairment in the locomotion domain

				3) Vitality - 'do you have any weight loss ≥5% within the past 6 months?' [Yes/No]. Those who scored "Yes" were considered as having an impairment in the vitality domain<br>4) Sensory capacity (vision) - according to ICOPE handbook. Those who answered "very poor/poor/not too well" were classified as having impairment in the sensory (vision) domain<br>5) Sensory capacity (hearing) - "Do you hear things clearly" with five responses (very poor/poor/not too well/fair/good/very good). Those who answered "very poor, poor, not too well" were classified as having an impairment in the sensory (hearing) domain<br>6) Psychological capacity - a) evaluative well-being 'Are you satisfied with your life?' [Yes/No]; b) hedonic well-being 'are you happy with your life?' {a Likert scale from 0 to 8, with 0 being the most unhappy and 8 being the happiest]; c) eudemonic well-being 'do you find purpose or meaning in your life' [Yes/No]. Those who answer 'no' for evaluative or eudemonic well-being or scored ≤3 (the lowest quartile) for hedonic well-being were considered as having an impairment in the psychological domain<br><br>The number of impaired IC domains ranged from 0 to 6, 0 representing no impairment and 6 representing impairments in all domains.
Zhao et al 2021	To estimate the status of IC and examine whether impairment in IC predicts subsequent 1-year disability in a representative community-dwelling Chinese older population.	A representative community-dwelling older population cohort.<br><br>n=7298<br><br>Inclusion criteria: 65 years and older<br><br>Exclusion criteria: Not specified	Not specified.	Five IC domains included:<br>1) Locomotion: Tinetti score (0 to 26). Those scored <24 was considered as having an impairment in locomotion<br>2) Vitality: Mini-Nutritional Assessment (MNA) scale (maximum score of 30). MNA score <24 was considered as an impairment in vitality<br>3) Sensory (vision and hearing impairments). Participants were asked if they experienced any recent decline in vision and hearing. A positive answer to a recent decline in vision or hearing impairments was considered as an impairment in sensory capacity<br>4) Cognition: using MMSE (maximum score of 30). MMSE <24 was considered as having impairment in cognition<br>5) Psychology: 15-item Geriatric Depression Scale (0 to 15 with higher score denotes worse performance). GDS-15 ≥8 was considered as having a psychological impairment.

				1=impaired; 0=not impaired
**WHO Clinical Consortium on Healthy Ageing 2019: report of Consortium meeting held 21-22 November 2019, Geneva, Switzerland.**				
China	The ICOPE pre-pilot project in China had three objectives: evaluation of ICOPE, standardization of interventions and evaluation of impact on outcomes for older persons.	Older persons aged ≥60 years.<br><br>n=3000	Not specified.	The symbol/slogan of ICOPE in China can be translated as "ICOPE will bring older people happiness"
France	Aims of the INSPIRE project included (i) Inspire basic science and animal cohort (e.g., understand the mechanisms and develop treatments to prevent the consequences of ageing: inflammation fibrosis, loss of metabolic-flexibility). (ii) Inspire translational and the clinical cohort (e.g. clinical care cohort: the evolution and optimal maintenance of IC (ICOPE). (iii) Digital community (e.g. establishment of a function monitoring centre from home – ICOPE digital tool). (iv) Establish an educational programme. (v) Establish a large bio-campus dedicated to healthy ageing.	Older persons aged more than 60 years (a group for those aged 40–60 years is being considered).<br><br>n=1000	Not specified.	The next steps will involve addressing how the ICOPE approach would be paid for, through small payments outlined in five steps
India	The goal of the pre-pilot project in India is to implement ICOPE as cornerstone for community-based care of older persons in India.		Older adults aged more than 75 years.<br><br>n=3000	The future plans include strengthening of The National Program for Health Care of the Elderly (NPHCE) with inclusion of ICOPE, and including ICOPE in geriatric services of the Health Wellness Centre under the Ayushman Bharat Scheme.
Costa Rica, Mexico	The aim of the study is to study the association between IC and likelihood of death. The study uses an IC index, which is a summary index built from the five ICOPE domains using a scale of 0 (severe deterioration) to 10 (optimal) points. CRELES aims to understand the impact of IC on primary outcomes on hospital admission, gait speed and grip strength, and secondary outcomes on care dependence and frailty status	Nationally representative sample.<br><br>n=1888	Not specified.	Not specified.
**WHO clinical consortium on healthy ageing 2020: report of consortium meeting held virtually, 18-19 November 2020. Geneva.**				

China	To test the readiness for China to introduce an innovative care model such as ICOPE through a prospective study, to study the sustainability and scalability of the model design to support healthy ageing-related policy, research and practice, so as to ensure that the health and care needs of older people can be met in a timely manner.	Those living in communities, aged 80 years and older with risk of declines in IC determined through ICOPE screening questions.<br><br>n= 2200 (25% intervention group; 75% control group)	Multiple stakeholders work together to provide person-centred care using the ICOPE handbook.	
France	1. Identifying biomarkers of ageing. INSPIRE will provide clinical and digital data as well as biospecimens from both human and animals, to foster the identification and validation of (a set of) biomarkers of ageing 2. Implementing IC-centred health care pathway for ageing (ICOPE care pathways). Beyond implementation of ICOPE care pathways, INSPIRE aims to foster investigations on both the biological changes related to healthy ageing and the development of new technologies and metrics to enable the continuous monitoring of the ageing process.	For the study of ageing biomarkers and IC: 1000 men and women, aged 20 years and older (no upper limit), mostly community-dwellers (some institutionalized people have been recruited as well)<br><br>For ICOPE implementation: older people using primary care services (community nurses, general practitioners, etc.)	Toulouse University Hospital Gérontopôle, the WHO Collaborating Centre for Frailty, Clinical Research and Geriatric Training	INSPIRE has 2 components:<br><br>For the study of ageing biomarkers and IC: Observational 10-year longitudinal study<br><br>For ICOPE implementation: Pragmatic implementation of ICOPE in the health care system
India	In India, the ICOPE implementation pilot study has three aims: i) to assess the effectiveness of adapting ICOPE guidance in rural settings; ii) aligning ICOPE to the existing primary care system for the care of older people; and iii) assessing health system requirements to implement ICOPE.	Not specified		Due to the low literacy rates of older people in this state, self-ICOPE screening by older adults in rural areas is not feasible. Screening and assessment by community health workers, however, is possible. Community-based data collection by house-to-house surveys will form the basis of interventions. Community health workers, case managers and navigators (the latter are people trained to assist patients in seeking the right level of care in hospital settings will help align health and social services and facilitate referral of rural older adults. The health care system will require the ICOPE screening tool is further adapted to the local context. The cognitive assessment will need the flexibility to be modified to suit colloquially used syntax and vocabulary, with the inclusion of Hindi Mental State Examination for the cognitive evaluation in step 2. This adaptation includes integrating ICOPE into rural India's local health care pathway, though no single solution will suit the entire country.

**WHO Integrated care for older people (ICOPE) implementation pilot programme: findings from the 'ready' phase. Geneva.**				
China	At all the study sites, the preparation phase focused on adapting or developing the ICOPE tools to be used during implementation; recruiting health and care workers and older people to participate in the studies; training health and care workers and building partnerships across sectors.<br><br>Each of the four sites made some adaptations to the tools ICOPE for their contexts.	n=874 participants<br><br>Mean age= 82.8 years (70–100 years), 61% female	1,711 health and care workers (1053 nurses, 245 pharmacists, 104 doctors and 20 post office workers (for Step 1 training); 410 nurses (for Step 2 to 4 training).<br><br>Ministry of Civil Affairs; National Health Commission; National Healthcare Security Administration; National Committee on Ageing; National Research Centre on Ageing; Beijing Bureau of Civil Affairs; Beijing Health Commission; Beijing Healthcare Security Administration; Chaoyang Elderly Care Service Centre Nineteen hospitals in Beijing; 109 health centres; 12 older people's care stations run by the government and 25 run by civil society organizations; WHO China office	A multimedia campaign through both traditional and social media was used at the Chaoyang site to recruit older participants. The multimedia campaign used to recruit participants<br>in Chaoyang also targeted health and care workers (it reached some half a million people in total). This case study also proactively engaged health centres and professional associations.<br><br>Over 22 000 health workers, including primary care physicians, nurses, rehabilitation therapists and social workers, showed interest in the study, and the team engaged over 200 partner organisations and facilities
France		N=10,903 participants,<br><br>Mean age=76 years (18–108 years) with 96% aged 60 or more), 61% female	Regional Union of Health Professionals; University Department of General Medicine; Occitanie Roussillon Federation of Healthcare Homes; multi-professional health homes; Information and Prevention Centre; Health Insurance Examination Centre; Postal Service; Haute-Garonne Departmental Council; Pension and Occupational Health Insurance<br>Fund; National Old Age Insurance Fund; several territorial professional health communities; town halls; seniors' residences	The study at the Occitanie in France has reported the numbers of participants receiving referrals to care among those 958 older people for whom data were uploaded in the FRAILTY-ICOPE database from and interventions to 374 participants (39%) for vision, including referring them to comprehensive eye care; 623 people (65%) on cognition, such as by referring them to a memory clinic or for cognitive stimulation; 396 (41.3%) for hearing care, including providing hearing aids; 838 older people (86.4%) on mobility, such as referring them to physiotherapy; 740 (77.2%) on nutrition, with dietary advice for example; and to 429 participants (44.8%) on mood, including to give them advice on their social environment.<br><br>1,711 health and care workers (1053 nurses, 245 pharmacists, 104 doctors) and 20 post office workers) were trained.<br><br>Digital tools included the ICOPE Monitor mobile app for screening to collect longitudinal data from screening every six

				months. The ICOPE Bot online tool for screening, mobile phone, tablet or laptop used a conversational robot to guide through the screening process. The FRAILTY-ICOPE database was used for storing, reviewing, and analysing data. There are alerts generated for health workers on data on participants' status, risks and follow up.
India		n= 451<br><br>Mean age= 68 years (65–98 years), 46% female	The team in Rajasthan worked with students at the school of public health with existing responsibilities in community health. The school was a collaborating partner in the pilot. All India Institute of Medical Science; Asian Centre for Medical Education, Research and Innovation; Rural Health Centre of State Medical and Health Services; Community leaders	The collaborating rural health centre in Rajasthan identified participants with the help of a word-of-mouth approach driven by members of the older community taking part in the recruitment strategy. ICOPE was viewed as a way for older people to access care tailored to their specific needs, something they had not seen before. The collaborating rural health centre in Rajasthan identified participants with the help of a word-of-mouth approach driven by members of the older community taking part in the recruitment strategy. ICOPE was viewed as a way for older people to access care tailored to their specific needs, something they had not seen before. Study period was January to May 2021.
Andorra		n=72 participants,<br><br>Mean age= 73 (65–92 years), 54% female	Ministry of Health; City Council of Canillo; national health-care system; older people's social clubs	NA
